# Combined Venlafaxine and Olanzapine Prescription in Women with Psychotic Major Depression: A Case Series

**DOI:** 10.1155/2011/856903

**Published:** 2011-04-04

**Authors:** Lucio Ghio, Werner Natta, Paola Rossi, Laura Peruzzo, Elisa Zanelli, Simona Gotelli, Filippo Gabrielli

**Affiliations:** Department of Neuroscience, Ophthalmology and Genetics, University of Genoa, 16100 Genova, Italy

## Abstract

Patients with psychotic major depression suffer prolonged duration and greater severity of illness, including an increased likelihood of recurrent episodes and resistance to conventional pharmacotherapies. They do not respond to placebo and respond poorly to antidepressant or antipsychotic monotherapy. On the other hand, as has been demonstrated, they do respond well to antidepressant and antipsychotic combination therapies. Different combinations of drugs were studied, but little is known up to now with regard to the combination of venlafaxine and olanzapine. The following paper presents three separate case studies of female patients suffering from psychotic unipolar major depression, all of whom were admitted to a psychiatric ward and successfully treated with a combination of venlafaxine and olanzapine.

## 1. Introduction

Psychotic major depression affects approximately 20% of patients hospitalised for major depressive disorder [1, 2] and although it is predominantly higher in elderly patients it has also been known to affect young people [3].

Patients with psychotic major depression suffer prolonged duration and greater severity of illness [2, 4], as has been confirmed by their higher total scores on the Hamilton Rating Scale for Depression (HAM-D), and in particular their depressive symptoms scores [5]. They have a shorter symptom-free interval, an increased likelihood of recurrent episodes, are at a higher risk of suicide, and show resistance to conventional pharmacotherapies [2].

Compared with nonpsychotic major depression, patients with psychotic major depression do not respond to placebo and respond poorly to antidepressants or antipsychotics alone. On the other hand, as has been demonstrated, they do respond well to the combination of antidepressants and antipsychotics [6]. 

This combination is, at the moment, the strongest recommendation regarding psychotic major depression patients by the American Psychiatric Association Practice Guidelines [7].

Early studies of the effectiveness of this combination of antidepressants and antipsychotics were related to the combination of tricyclic antidepressants and conventional antipsychotics. Despite the positive results obtained, the use of these molecules has been reduced due to an undesirable high rate of adverse effects [8, 9]. 

Recent data suggests that the combination of SSRI antidepressants and atypical antipsychotics could prove as effective as the combination of tricyclic antidepressants and conventional neuroleptics [2, 6, 10].

Among atypical antipsychotic drugs olanzapine is the most studied in cases of major depression with psychotic symptoms. Results show efficacy in such cases, suggesting that olanzapine may have antidepressant properties [11–13].

With regard to new generation antidepressants, the majority of studies undertaken have focused on SSRIs, particularly fluoxetine [2]. 

We are not aware of any current study which has previously considered the combination of olanzapine and venlafaxine where psychotic depression is concerned. 

The existing case reports regarding the aforementioned combination are, to our knowledge, specifically aimed at the treatment of treatment-resistant depression [13, 14]. 

It has been suggested that randomised double-blind trials are “almost impossible” when evaluating treatment for psychotic major depression due to difficulty in obtaining informed consent [4]. Such trials are no longer ethically justified due to a patient's high risk of suicide; therefore, studying the effects of combining new drugs in single patients may prove useful.

As a result we have chosen to present three case reports involving patients suffering from psychotic unipolar major depression, all of whom were admitted into a women's psychiatric ward and all of whom were successfully treated with a combination of venlafaxine and olanzapine.

**Case A**

Mrs. A is 65 years old, suffering recurrent major depression, which began 10 years ago following her retirement, and which has since led to repeated hospitalisations in a psychiatric clinic as a result of severe depressive episodes with melancholic psychotic symptoms.

The patient has followed different psychopharmacological treatments, with the prescription of SSRIs and typical neuroleptics, which, over time, have resulted in an onset of extrapiramidal iatrogenic symptoms, at present unresolved.

The current hospitalisation was determined by a depressive episode which was characterised by depressed mood, psychomotor retardation, complete loss of autonomy, and recurrent thoughts of suicide. Delusions including persecution and inappropriate guilt were also present as well as auditory and visual hallucinations.

At admission, the patient had a HAM-D score of 30 and presented cognitive impairment and slowdown. An MMSE was administrated, with a score of 29.

Routine blood tests, including thyroid tests, did not show any relevant change and the patient's ECG was normal. The patient was affected by essential hypertension which was being controlled with tiazidic diuretics. Results from a cerebral TC scan were negative. 

During the initial phase of the patient's two-month treatment, she received a daily dose of diazepam (8 mg), clomipramine (maximum dose of 250 mg) and haloperidol (6 mg). There was no positive response shown to this initial phase of treatment, rather the patient worsened to a complete psychomotor arrest which in turn led to enteral nutrition and urinary catheterisation.

After one-month a new daily treatment was, therefore, administered, this consisted of intravenous lorazepam (8 mg), and venlafaxine (150 mg) in association with olanzapine (10 mg).

After 10 days the patient had begun to show her first signs of a progressive improvement, returning to feed herself and carrying out bodily functions independently.

After four weeks of the patient's second phase of treatment her HAM-D scale score went down to 7 and the patient was discharged.

The combination of venlafaxine and olanzapine (which was maintained following the patient's discharge) was well tolerated, with the exception of nausea during the first three days of treatment which spontaneously disappeared. No changes in the patient's blood pressure were noted.

The patient was discharged from this particular period of hospitalisation two years ago. Since that discharge she has had only one other brief admission on account of a voluntary suspension of therapy. She continues to maintain a satisfactory clinical balance at follow-up assessments.

**Case B**

Mrs. B is a 72 years old, admitted for the first time in her life at a psychiatric division. The patient's symptoms included depressed mood, insomnia, anhedonia, fatigue, decreased appetite (with weight loss) and heightened obsessive traits.

The most troubling symptom for the patient was the occurrence of simple auditory hallucinations, such as undefined, though alarming, noises. Delusional ideas of ruin and feelings of helplessness were also present.

The initial differential diagnosis at the clinical exam had to take into account the possibility of an onset of dementia, despite the absence of memory or orientation disorders, as the patient had no prior history of mood disorders. However, the results of a cerebral TC scan (with contrast enhancement) showed no sign of cerebral atrophy nor any significant vascular lesions. The patient's MMSE and HAM-D scores were reported as 25 and 20, respectively.

The HAM-D showed the highest scores for items such as weight loss, anxiety/somatisation, motor retardation and sleep disturbances.

Routine blood tests were within range, including thyroid function.

The diagnosis was a severe depressive episode with mood-congruent psychotic features.

We decided to administer a daily treatment of venlafaxine (150 mg) and olanzapine (5 mg). We also chose to include a daily dose of mirtazapine (15 mg) in order to improve the patient's loss of appetite, and delorazepam (0.5 mg) to treat her insomnia.

Within the first days of hospitalisation the patient's psychotic symptoms had disappeared alongside her insomnia. The patient's mood showed some improvement following the second week of treatment, allowing for the patient's discharge after only 16 days.

One month after discharge the patient's follow-up assessment showed a HAM-D score of 10. This included a 50% decrease in all areas with the exception of anxiety/somatisation for which there was a minimal decrease. The patient's MMSE score was 28.

**Case C**

Mrs. C is a 59 years old, who has had several admissions to hospital for recurrent major depression disorder.

After a long period of hospitalisation in a different psychiatric division, the patient was discharged with medical therapy consisting of high doses of sertraline (300 mg per day), alprazolam (2 mg per day), trazodone (75 mg per day) and flurazepam (30 mg per day). After only seven days the patient was again admitted to hospital for attempted suicide (lacerated veins) this time to our psychiatric clinic. The motivation for the suicide attempt was a reported worsening of mood, as well as delusions of poisoning and auditory hallucinations, both of which were noted for the first time in the patient's medical history. 

A year before hospitalisation in question, the patient had suffered a cerebral stroke and was therefore following a prescribed treatment of anticoagulants and statins. She was also affected by mild vascular hypertension which, in turn, was being treated with ACE inhibitors.

The results of the patient's neurological clinical exam showed a mild strength deficit at upper and lower left limbs, resulting from the cerebral stroke. The patient's cerebral TC scan was negative, as were her routine blood tests.

The patient's HAM-D score was 28, with higher scores related to anxiety/somatisation, cognitive disorders and sleep disorders items.

This particular episode for which the patient was admitted was treated as a new psychotic major depressive episode; however, we considered the possibility that the prescribed high dosage of sertraline may have induced the patient's psychotic symptoms (although rare, psychotic symptoms through SSRI therapy have been documented) [15, 16].

During the first seven days of hospitalisation we reduced, and then interrupted, the patient's intake of sertraline and trazodone. These were substituted with venlafaxine (225 mg per day) and olanzapine (5 mg per day) together with alprazolam (2 mg per day) and flurazepam (30 mg per day), with which the patient was already being treated.

Due to the fact that the patient had previously suffered a cerebral stroke the patient provided us with her informed consent in order to allow us to administer olanzapine.

The patient showed a prompt remission of symptoms and was subsequently discharged after 18 days.

The patient's HAM-D score at discharge was 8, showing a reduction of over 75% in all areas.

At the patient's one-month follow-up assessment a good clinical balance was being maintained and there were no signs of side effects caused by the drugs administered. As a precaution, the dosage of olanzapine was gradually reduced and finally suspended two-months after discharge, whereas the treatment with venlafaxine continues to the present day.

## 2. Discussion

These three case reports seem to demonstrate the positive effects of a venlafaxine and olanzapine combination in the treatment of psychotic major depression.

What is of particular relevance is the promptness of clinical response; two of the patients in our case studies being discharged in just over a fortnight and the third patient responding to the combination after one-month.

This promptness is particularly interesting, when taking into consideration the symptoms of psychotic major depression which include greater severity, greater incapacitation and prolonged periods of illness when compared to nonpsychotic major depression.

The efficacy of remission was similar in all three cases: both with the patients suffering recurrent depression, who had previously undergone several other treatments in their medical history, as with the patient suffering her first episode.

Furthermore all cases presented showed a good response to medium-low dosage (from 150 mg to 225 mg per day of venlafaxine and 5 mg to 10 mg per day of olanzapine). This might also explain the almost complete absence of side effects.

In particular the medium-low dosage of venlafaxine administered to the two patients (A and C) affected by hypertension had no influence on their respective parameters (an increase in blood pressure being a dose-related effect of venlafaxine).

Bearing in mind the study carried out by Zanardi et al. [17], which considers venlafaxine 300 mg per day an effective treatment for psychotic depression, we suggest that a combination with olanzapine may result in a useful option for those patients suffering psychotic major depression and hypertension.

With regard to the duration of treatment, it is not yet clear how long the antidepressant and antipsychotic association should last [3]. 

The fact that the patient described in Case A relapsed after an episode of nonadherence to therapy, two years after her initial discharge seems to indicate, in accordance with recent literature [18], the need for long-term treatment where antipsychotic and antidepressant combinations are prescribed, including cases in which the patient is suffering their first episode of psychotic major depression.

On the other hand in case C the precautionary suspension of olanzapine has not led to any clinical relapse though the venlafaxine dosage has not been modified (it has now been seven months since the patient's discharge). In this case we maintain the belief that sertraline may have caused the onset of the patient's psychotic symptoms.

Overall the association of venlafaxine and olanzapine in psychotic major depression has demonstrated efficacy, at least as much as the most investigated combination, between fluoxetine and olanzapine.

The data still need to be confirmed through clinical trials; however, there are difficulties in carrying out such studies given that patients are often admitted when they are in a critical condition and, as such, unable to provide an adequate, informed consent (e.g., patients who are in a catatonic state or who are affected by delusions of persecution). 

The resulting mechanism from this combination of venlafaxine and olanzapine remains, as yet, unclear. It could be related to the specific mechanisms of olanzapine and venlafaxine, and their pharmacodynamic interaction. Olanzapine is an antagonist and binds to serotonin (5HT_2A/2C_) and dopamine (D_1–4_) receptors, and thus may combine AD and AP effects in a single compound. Furthermore, olanzapine could increase the effect of serotonin antagonism in venlafaxine, likewise in fluoxetine. However venlafaxine, unlike fluoxetine, has a dual mechanism of action including a noradrenergic property, which may explain its more rapid and effective results in psychotic major depression patients.

##  Conflict of Interests 

The authors declare that they have no Conflict of Interests.
